# Early detection of Citrus Huanglongbing by UAV remote sensing based on MGA-UNet

**DOI:** 10.3389/fpls.2025.1503645

**Published:** 2025-05-06

**Authors:** Naibo Ye, Wenyong Mai, Feng Qin, Sen Yuan, Bo Liu, Zaiyuan Li, Conghui Liu, Fanghao Wan, Wanqiang Qian, Zhongzhen Wu, Xi Qiao

**Affiliations:** ^1^ Guangdong Laboratory of Lingnan Modern Agriculture, Genome Analysis Laboratory of the Ministry of Agriculture and Rural Affairs, Agricultural Genomics Institute at Shenzhen, Chinese Academy of Agricultural Sciences, China of Agricultural Sciences, Shenzhen, China; ^2^ Guangzhou City Key Laboratory of Subtropical Fruit Trees Outbreak Control, Institute for Management of Invasive Alien Species, Zhongkai University of Agriculture and Engineering, Guangzhou, China

**Keywords:** Citrus HuangLongBing, citrus greening, UAV, multispectral images, deep learning, generative model

## Abstract

Citrus Huanglongbing (HLB), also known as citrus greening, is a severe disease that has caused substantial economic damage to the global citrus industry. Early detection is challenging due to the lack of distinctive early symptoms, making current diagnostic methods often ineffective. Therefore, there is an urgent need for an intelligent and timely detection system for HLB. This study leverages multispectral imagery acquired via unmanned aerial vehicles (UAVs) and deep convolutional neural networks. This study introduce a novel model, MGA-UNet, specifically designed for HLB recognition. This image segmentation model enhances feature transmission by integrating channel attention and spatial attention within the skip connections. Furthermore, this study evaluate the comparative effectiveness of high-resolution and multispectral images in HLB detection, finding that multispectral imagery offers superior performance. To address data imbalance and augment the dataset, this study employ a generative model, DCGAN, for data augmentation, significantly boosting the model’s recognition accuracy. Our proposed model achieved a mIoU of 0.89, a mPA of 0.94, a precision of 0.95, and a recall of 0.94 in identifying diseased trees. The intelligent monitoring method for HLB presented in this study offers a cost-effective and highly accurate solution, holding considerable promise for the early warning of this disease.

## 1 Introduction

Citrus Huanglongbing (HLB) is a bacterial disease. It poses a serious threat to the health and yield of citrus trees. The disease spreads primarily through psyllids. It can also spread via infected seedlings and grafting practices. (Halbert and Manjunath, 2004) Symptoms of HLB include leaf yellowing and uneven fruit ripening. In severe cases, it often leads to tree mortality. The disease is prevalent across Asia, Africa, South America, and North America. It has profound economic implications for the citrus industry. For example, in 2017, the citrus industry in Guangdong Province, China, incurred losses exceeding $500 million due to HLB. Currently, there is no cure for HLB. Once a tree is diagnosed, it must be removed. Therefore, early detection and monitoring are critical for managing the disease.

However, early detection of HLB remains a significant challenge for citrus growers. Presently, methods such as field visual inspection (Garcia-Figuera et al., 2021), electron microscopy, grafting diagnosis (Lin et al., 2017), and PCR testing are employed (Li et al., 2006). Field visual inspection requires experts to visit citrus orchards to observe potentially infected trees. This process depends heavily on the inspectors’ experience. Electron microscopy can reveal the pathogen’s structure and is useful in detecting HLB. However, both field inspection and electron microscopy have low accuracy in identifying early-stage HLB. Grafting diagnosis involves attaching branches suspected of HLB infection to healthy indicator plants. Observing pathological symptoms takes 6 to 10 months or more. PCR testing identifies pathogen DNA sequences in the tree and compares them to HLB-specific sequences to confirm infection. Yet, both grafting diagnosis and PCR testing are expensive and difficult to scale. This study proposes an innovative intelligent detection method for HLB. It leverages images captured by unmanned aerial vehicles (UAVs) combined with convolutional neural networks (CNNs). This approach offers faster identification and lower detection costs than traditional methods.

In recent years, machine learning has become the leading approach for image recognition. Various mature solutions exist for applying machine learning in HLB detection (Lan et al., 2020). Some studies have used machine learning techniques like linear regression and support vector machines for HLB detection. However, challenges persist. Indistinct disease characteristics and blurred image boundaries hinder recognition accuracy. In this study, we introduce MGA-Unet, a deep learning model based on the U-net architecture specifically designed for image segmentation. MGA-Unet excels at distinguishing edge information and fine details. This makes it well-suited for tasks that require precise feature differentiation, such as early HLB detection.

Building a robust dataset is essential for the intelligent detection of HLB. Previous research often utilized handheld cameras for close-range image acquisition (Qiu et al., 2022), but this approach is inefficient and unsuitable for large-scale data collection. UAV remote sensing technology, a burgeoning tool in plant protection, offers rapid image collection and analysis capabilities (Bendig et al., 2015). UAVs can be equipped with high-resolution, multispectral, or hyperspectral cameras to capture imagery. Recent studies have shown the applicability of high-resolution camera images captured by UAVs in HLB detection (Garza et al., 2020). However, due to the subtlety of early HLB symptoms, the use of high-resolution imagery for dataset construction presents limitations in recognition accuracy. This study investigates the use of multispectral camera imagery as a dataset for early HLB detection. Meanwhile compares its performance with high-resolution images.

A model’s performance depends not only on algorithmic optimization but also on the quality of the dataset. In the context of early HLB detection, the proportion of diseased citrus trees in the dataset is relatively small. Deep learning models require a substantial amount of both positive and negative samples, and imbalanced datasets can adversely affect model training. Addressing the issues of small data size and class imbalance can significantly enhance model performance. Traditional data augmentation methods can increase dataset size but it doesn’t fundamentally change the dataset.

This study employs a specialized data augmentation strategy by leveraging the DCGAN model, a variant of generative adversarial networks (GANs), to address the small data size and class imbalance in the HLB dataset. This approach enhances both the robustness of the dataset and the performance of the subsequent machine learning models. Specifically, we introduce an MGA-Unet model designed for early detection and identification of HLB, incorporating channel attention and spatial attention with optimized skip connections. We also utilize multispectral images acquired via an unmanned aerial vehicle (UAV) platform in conjunction with convolutional neural networks (CNNs) for rapid early detection of HLB, and compare recognition performance between high-resolution image data and multispectral image data. Furthermore, the DCGAN generative model is employed for data augmentation in HLB image segmentation, with recognition performance evaluated both with and without DCGAN augmentation.

## 2 Materials and methods

### 2.1 Data sources

**Figure 1:**
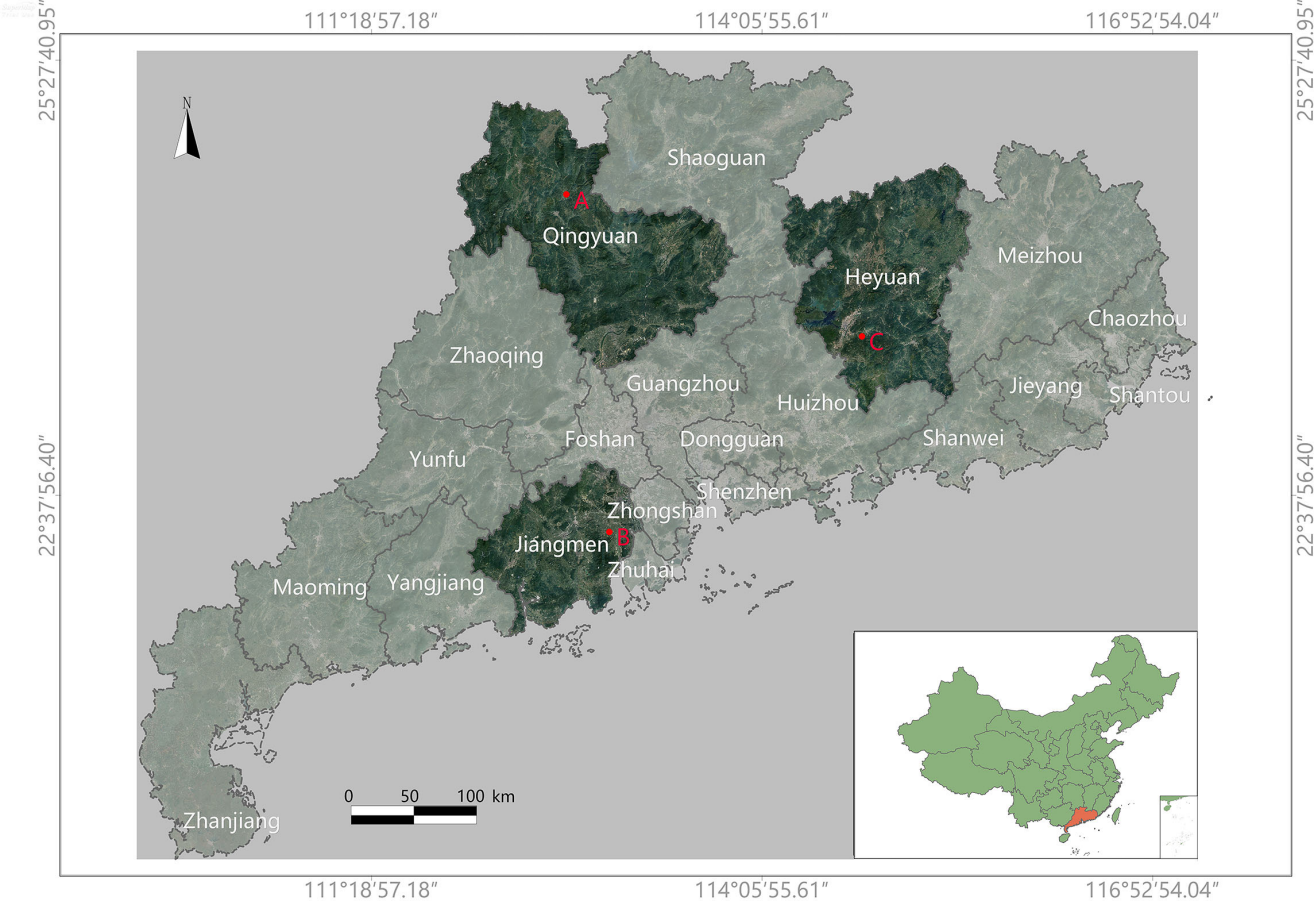
This is a map of Guangdong Province, China. The specific data collection locations are marked on the map as ‘A’, ‘B’, and ‘C’. The collection times and corresponding coordinates (latitude and longitude) for each location are as follows: **(A)** Yingde City, Qingyuan (2023.01.04), covering 7581m^2^, at coordinates (112.89928897, 24.40084808); **(B)** Xinhui District, Jiangmen (2023.02.18), covering 3740m^2^, at coordinates (112.97968806, 22.40470336); **(C)** Zijin County, Heyuan (2023.04.25), at coordinates (114.98817667, 23.43008256).

The dataset was acquired from various citrus production bases in Guangdong Province, China. Guangdong is located in southern China, characterized by a subtropical monsoon climate, making it suitable for citrus cultivation (Luo et al., 2017). To ensure high-quality data, image capture was conducted during periods with minimal solar radiation interference. The specific data collection sites are shown in **Figure 1**.

A DJI Mavic 3M drone was used for data collection. It offers up to 43 minutes of flight time per mission and can cover up to 2 square kilometers per flight. The drone was equipped with both a high-resolution camera and a multispectral camera. This setup allowed for the simultaneous capture of two image types during a single flight. The multispectral camera was capable of capturing four spectral bands: Red Edge (RE), Red (R), Near-Infrared (NIR), and Green (G). The camera parameters are provided in detail in **Table 1**.

**Table 1:** DJI Mavic 3M camera parameters.

Item	RGB Camera	Multispectral Cameras
Max Image Size	5280 x 3956	2592 x 1944
Photo Pixel	20 MP	5 MP
Image Sensor	4/3 CMOS	1/2.8-inch CMOS
Equivalent focal length	24 mm	25 mm
Aperture	f/2.8 to f/11	f/2.0

### 2.2 Labeling disease data methodology

#### 2.2.1 Sample collection

Samples of citrus leaves suspected to be infected with Huanglongbing (HLB) were collected from a variety of citrus species, including *Citrus reticulata* ‘Chachiensis’, *Citrus reticulata* ‘Shatangju’, *Citrus maxima*, and *Citrus sinensis*. Trees were selected and marked as individual units within predefined areas. For each tree, 1–2 leaves were collected from 12 distinct locations corresponding to four directions (east, south, west, and north) at three canopy heights (upper, middle, and lower layers). The collected leaves were stored in sample bags and immediately transported to the laboratory, where they were stored at -20°C for further processing.

#### 2.2.2 Total DNA extraction from citrus leaves

Total genomic DNA was extracted from the collected citrus leaves using an automated nucleic acid extraction and purification system. A magnetic bead-based plant genome extraction kit was used for this purpose. To prepare for extraction, 12 leaf samples were pooled, and 0.1g of main vein tissue was placed into a 2mL centrifuge tube containing grinding beads. This was followed by mechanical disruption using an MP FastPrep-24 homogenizer. After disruption, the appropriate reagents were added following the kit protocol, and the sample was processed through the extraction and purification system. The resulting DNA was transferred into 1.5mL centrifuge tubes and stored at -80°C for long-term preservation.

#### 2.2.3 Real-time quantitative PCR detection of HLB pathogen

To detect Huanglongbing (HLB), a real-time quantitative PCR (qPCR) assay utilizing a dual-probe strategy was employed. The reaction mixture was prepared using the Premix EX Taq™ (Probe qPCR) kit (RR390A, TaKaRa). The specific primers used are listed in **Table 2**.The qPCR reaction mixture consisted of 10 µL of premix solution, 0.4 µL of 10 µM forward and reverse primers, 0.3 µL of 10 µM probes, and 2 µL of the extracted DNA template. The final volume was adjusted to 20 µL with ddH2O. Amplifications were performed on a Roche LightCycler 480 II qPCR instrument under the following cycling conditions: initial denaturation at 95°C for 5 minutes, 58°C for 30 seconds (ramp rate of 2.2°C/s) and followed by 45 cycles of 95°C for 5 seconds. The denaturation phase was set at 95°C for 10 seconds, 40°C for 30 seconds (ramp rate 2°C/s), followed by 80°C for 30 seconds (ramp rate 0.06°C/s). Annealing was carried out at 50°C for 30 seconds (ramp rate of 2.2°C/s). Positive controls, negative controls, blank controls, and standard curve controls were included in the assay, were included to ensure the accuracy and reliability of the results.

**Table 2:** Primer sequence list.

Name	Sequence(5′-3′)
HLB-F	ACGCTGGCGGCAGGCTAA
HLB-R	GTAGATTCCTACGCGTTACTCA
HLB-P	FAM-TCGAGCGCGTATGCGAAT-BHQ
COX-F	GTATGCCACGTCGCATTCCAGA
COX-R	GCCAAAACTGCTAAGGGCATTC
COX-P	TET-ATCCAGATGCTTACGCTGG-BHQ-2

In this study, the location of each tree was recorded during leaf collection. If any leaf sample from a particular tree tested positive for the HLB pathogen via PCR detection, that tree was labeled as diseased. Conversely, if none of the sampled leaves from a tree tested positive, the tree was labeled as healthy. These labeled data points are utilized in the image preprocessing stage. If a tree is labeled as “diseased,” this study classifies the entire tree into the diseased category. Due to the orthophoto imagery being captured from an unmanned aerial vehicle (UAV) perspective, the spatial scale and viewpoint only allow the detection of disease characteristics at the canopy or population level. Moreover, the overhead viewpoint captures only the top portions of the trees; therefore, each tree is treated as a whole entity for classification purposes.

### 2.3 Image preprocessing

#### 2.3.1 Dataset processing

**Figure 2:**
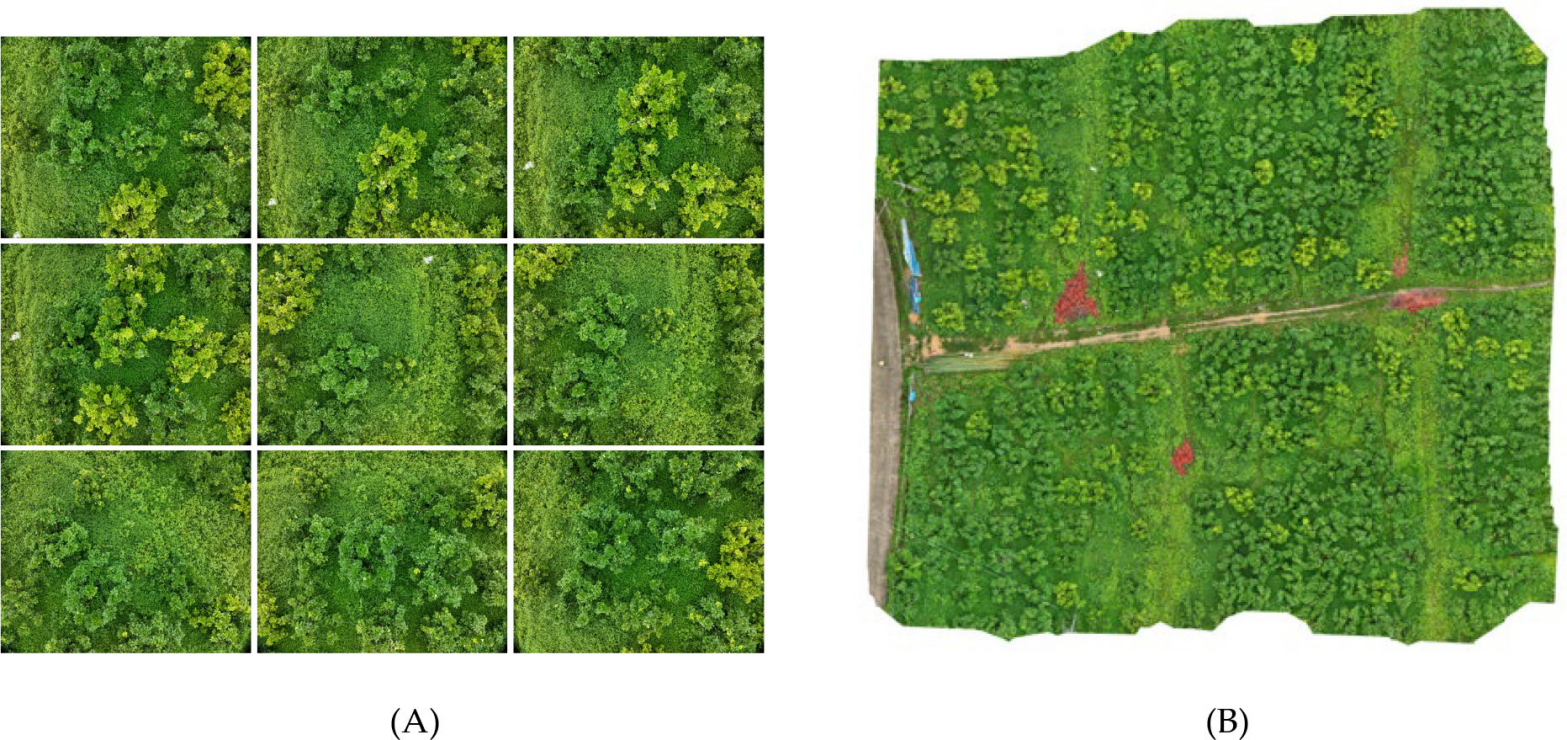
This is a description of the image stitching process. **(A)** shows a series of consecutive images captured by the drone, with some overlapping pixels between the adjacent images. **(B)** illustrates the result of stitching these images together in the order and positions they were captured, removing the overlapping pixel regions to form a composite image.

The raw images captured by the drone’s cameras require preprocessing before they can be utilized for analysis. To maintain image continuity and completeness, it is necessary to address the overlapping regions at the edges of drone-captured images (Wang et al., 2022). These images are sequentially stitched together to form larger, continuous datasets for subsequent processing. The methodology is illustrated in **Figure 2**. His study also compares the efficacy of high-resolution images and multispectral images in detecting HLB. High-resolution images are ready for use without any additional processing. In contrast, multispectral images require synthesis across various spectral bands to enhance their utility in detection tasks.

#### 2.3.2 Multispectral image preprocessing

In the context of HLB detection, color and texture features are more critical than shape features. Multispectral images are particularly effective in accentuating color characteristics, which in turn enhance the clarity of texture features at object boundaries. When employing multispectral data, deep learning models can more effectively learn the pathological features associated with HLB. However, single-band multispectral images are limited in the information they provide. By synthesize multispectral images in the following bands: Red Edge (RE), Red (R), Near-Infrared (NIR), and Green (G). This can highlight more useful information.

- G (Green): The Green band, centered around 560 ± 16nm, is particularly sensitive to plant photosynthesis.
- R (Red): The Red band, centered around 650 ± 16nm, is sensitive to chlorophyll absorption. Healthy vegetation exhibits lower reflectance in this band, making it useful for distinguishing vegetation from soil.
- RE (Red Edge): The Red Edge band, centered around 730 ± 16nm, lies between the red and near-infrared bands. It is highly effective for differentiating between vegetation types and assessing vegetation health due to the significant reflectance changes observed in healthy vegetation.
- NIR (Near-Infrared): The Near-Infrared band, centered around 860 ± 26nm, is characterized by high reflectance in healthy vegetation, while water bodies almost completely absorb this wavelength.

**Figure 3:**
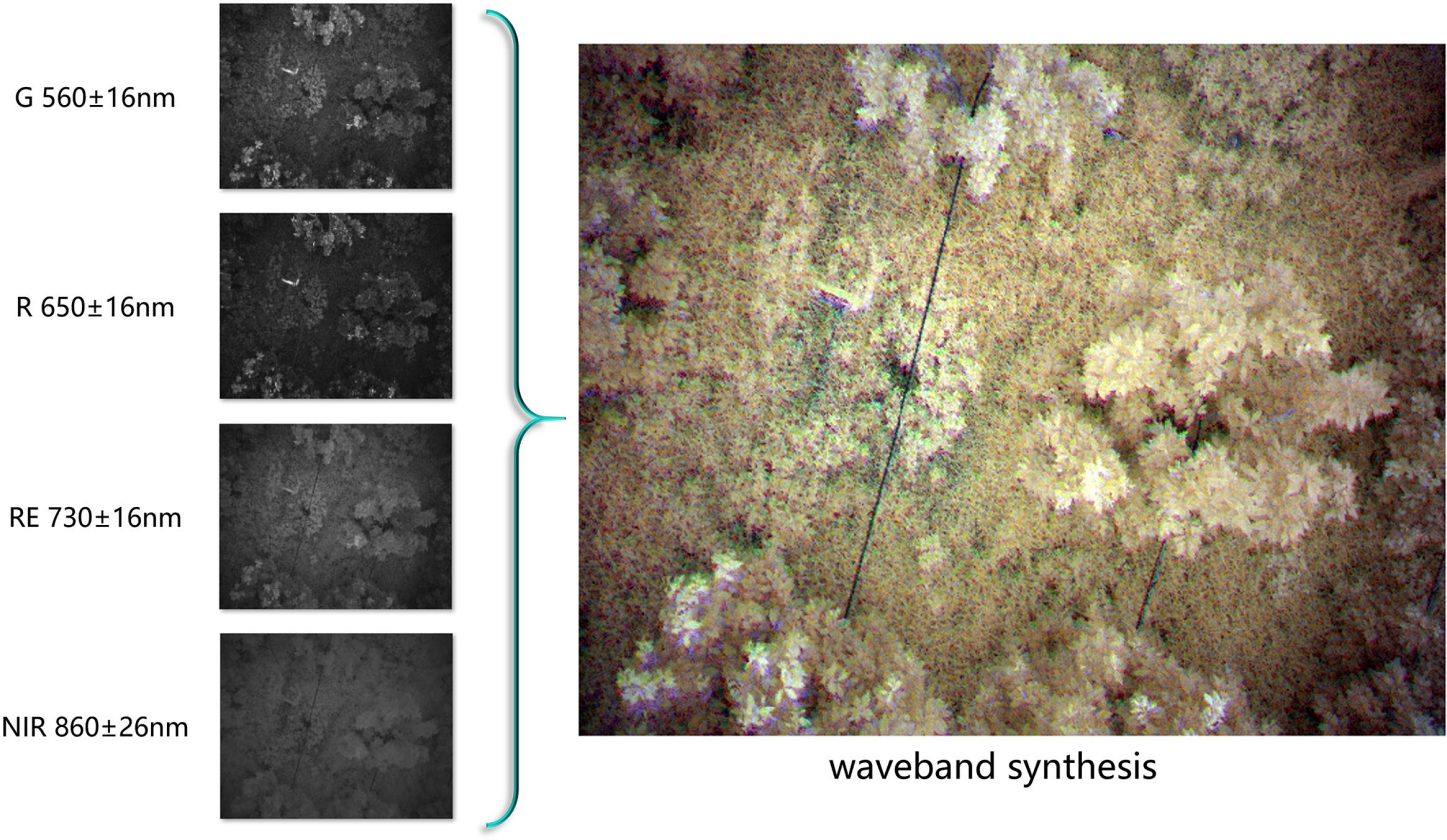
Band synthesis requires combining several grayscale multispectral images, captured at the same location but across different spectral bands, into a single false-color image. In this image, different colors are assigned to various multispectral bands, allowing the image to simultaneously contain information from multiple bands while rendering certain features visible to the human eye.

There are two primary methods for processing multispectral images:

1. Vegetation Index: The vegetation index is a widely used approach for processing multispectral data, with common indices including NDVI, GNDVI, SAVI, NIR-R, G/R, and NIR/R (Garcia-Ruiz et al., 2013). NDVI, which relies on the reflectance of the red and near-infrared bands, is often used to detect vegetation and assess chlorophyll content. It is effective in highlighting vegetative areas, as there is a positive correlation between chlorophyll content and NDVI values (Jones et al., 2007). NDVI can also reflect differences in vegetation water content and nitrogen levels. However, for HLB detection, the differences between healthy and HLB-affected trees are not always significant when using vegetation indices.
2. Color Synthesis: By combining data from different wavelength channels of multispectral images, a synthesized false-color image can be produced (Nekhin et al., 2022). False-color images map information from non-visible spectrum bands (e.g., infrared) onto the visible spectrum’s color channels (red, green, blue), creating a visual representation that emphasizes features from different bands. In the context of HLB detection, where leaf yellowing is a key indicator, it is crucial to clearly distinguish between yellow and green in the images. This study utilizes multispectral color synthesis techniques for image preprocessing to enhance the visibility of these critical features. The implementation method is shown in **Figure 3**.

### 2.4 Data augmentation

A high-quality dataset is pivotal to the successful training of a model (Gharawi et al., 2022), as imbalances or insufficient size can negatively impact the training outcomes. When a dataset is imbalanced, it can skew the model’s learning process (Zhang et al., 2019), and if the dataset is too small, the model is prone to overfitting (Fang et al., 2023). Data augmentation techniques should be tailored to the specific image categories and their characteristics. However, a fundamental principle is to introduce as much variation as possible without altering the original semantic information. Data augmentation enhances model performance. In some instances, it also reduces the need for extensive data labeling efforts.

Generally, data augmentation offers three key benefits:

1. It enhances the model’s generalization capabilities, enabling better performance on unseen datasets.
2. It reduces overfitting by helping the model learn essential image features, thereby preventing the model from becoming too specialized on the training data.
3. It effectively expands the dataset, increasing its size and addressing class imbalance.

#### 2.4.1 Traditional data augmentation methods

Geometric transformations are a common technique, generating new images by repositioning pixel values through rotation, flipping, scaling, translation, and cropping (Shorten and Khoshgoftaar, 2019). These methods preserve the fundamental shape of the images while altering their orientation and position.

Techniques like sharpening and blurring modify image details and edge contrasts to achieve various effects. Sharpening enhances details, while blurring diffuses pixel values, softening edges and diminishing detail prominence. These methods can either emphasize or downplay semantic features in an image.

Noise perturbation involves adding random or fixed noise patterns to images. Noise can significantly impact object recognition in deep learning, as even small amounts of noise can lead to misclassification. However, appropriately added noise can improve the robustness of image processing algorithms and increase dataset diversity.

While traditional data augmentation methods can improve a model’s generalization, they have limitations. These methods generally apply only basic transformations, resulting in augmented images that remain fundamentally similar to the originals. To overcome these limitations, this study also employs Generative Adversarial Networks (GANs) to generate new image data, further optimizing data augmentation.

#### 2.4.2 Generative model-based data augmentation

Generative models create new data based on existing datasets. These synthetic data are not real but are generated by capturing and replicating the characteristics of the training set. Although these generated images share features with the real dataset, they do not exactly match any real data. Such synthetic data are invaluable, particularly in fields like plant identification, where data collection is constrained by specific growth periods. Missing data collection during these periods can lead to long delays in data acquisition. In plant pathology identification, where data on diseased specimens may be scarce, healthy images can be used to generate images of diseased specimens. Generative models can also be employed to produce synthetic data that exclude sensitive information. This capability makes them useful in scenarios requiring confidentiality. Additionally, they can be used to remove watermarks from images (Yang et al., 2020; Huang and Cao, 2023). Generative models thus offer a solution to the challenge of acquiring difficult-to-obtain data.

In this study, the Deep Convolutional Generative Adversarial Network (DCGAN), a type of GAN-based unsupervised learning model, was employed. DCGAN enhances model performance through adversarial training between a generator and a discriminator. The generator’s goal is to create realistic synthetic data that the discriminator cannot distinguish from real data. Meanwhile, the discriminator’s objective is to differentiate between real and synthetic data. Through this adversarial process, both the generator and discriminator progressively improve. When the model reaches Nash equilibrium, the discriminator can no longer reliably distinguish between real and generated data. At this point, the model gains the ability to generate data that closely resembles real images.

Although GANs have shown great promise in generating data through adversarial training, they initially faced challenges such as training instability and mode collapse. Deep Convolutional Generative Adversarial Network (DCGAN) addresses these issues by significantly improving the quality and stability of the generated images. The training process of DCGAN is similar to that of traditional GANs but includes convolutional layers and design optimizations. These improvements enhance network stability and make the training process more efficient. In DCGAN, the discriminator uses convolutional layers to gradually extract feature maps. Meanwhile, the generator employs transposed convolutional layers (also known as deconvolution layers) to upsample and reconstruct the feature maps into images. Typically, each layer includes batch normalization and activation functions like ReLU.

In this study, data augmentation using DCGAN was applied exclusively to the minority class—specifically, diseased tree samples. Since our samples were collected during the early stages of HLB disease, the number of diseased tree samples was significantly smaller compared to healthy ones, resulting in severe class imbalance. To address this, the DCGAN was trained on each cropped image containing complete diseased trees, generating additional synthetic samples to enrich the dataset of diseased tree images.

### 2.5 Machine learning models

Numerous studies have utilized machine learning models for plant pathology recognition, employing techniques such as Support Vector Machines (SVM) (Tomar and Agarwal, 2016), K-Nearest Neighbors (KNN) (Ambarwari et al., 2016), Decision Trees (Samal et al., 2002), and Random Forests (Polyakova et al., 2023). While these methods have demonstrated success, early identification of plant diseases remains challenging due to the small size and subtlety of affected areas. To further improve recognition accuracy, deep convolutional neural network (CNN) models are increasingly necessary (Chen et al., 2023).

#### 2.5.1 Deep learning models

Some researchers have employed YOLOv5 as a baseline model, achieving a micro-F1 score of 85.19% (Qiu et al., 2022).These methods primarily rely on datasets of close-range images captured with handheld cameras (Deng et al., 2016; Barman et al., 2020; He et al., 2022; Qiu et al., 2022). Drone-captured images pose unique challenges, such as resolution and angle constraints. Therefore, more specialized models are necessary to achieve effective recognition.

#### 2.5.2 MGA-UNet models

The U-Net model, originally developed for medical image segmentation (Ronneberger et al., 2015), consists of an encoder (Contracting Path) and a decoder (Expanding Path), connected through skip connections. This progressive encoding-decoding structure allows U-Net to extract features at multiple levels. It captures edge and texture details at the pixel level while also considering shape and position on a global scale. Despite its strong performance, the traditional U-Net model may struggle with understanding global contextual semantics in complex scenarios, leading to potential feature loss. To overcome these limitations, this study introduces the MGA-UNet model. It enhances the U-Net architecture by incorporating channel attention and spatial attention mechanisms within the skip connections to optimize feature transmission.

The encoder (contracting path) reduces the spatial dimensions of the image while increasing the depth of the feature maps. This process facilitates the extraction of high-level features. It consists of convolutional layers followed by pooling layers. Each convolutional layer comprises two 3x3 convolutional kernels paired with ReLU activation functions, designed to capture local features from the image. Pooling layers follow the convolutional layers, reducing the size of the feature maps. After each pooling operation, the spatial dimensions of the image are halved, and the number of channels is doubled. This approach reduces computational complexity and the number of parameters.

The bottleneck serves as the connection between the encoder and decoder, containing two 3x3 convolutional layers and ReLU activation functions to extract deeper features. Skip connections are essential as they concatenate feature maps from corresponding layers in the encoder and decoder. This helps preserve information that may have been lost during encoding and aids in the image reconstruction process within the decoder.

The decoder (expanding path) progressively increases the spatial dimensions of the image while reducing the depth of the feature maps, ultimately restoring the image to its original size. The decoder comprises up-sampling and convolutional layers, where up-sampling is achieved through 2x2 transposed convolution, effectively doubling the image dimensions. This is followed by two 3x3 convolutional kernels and ReLU activation functions.

**Figure 4:**
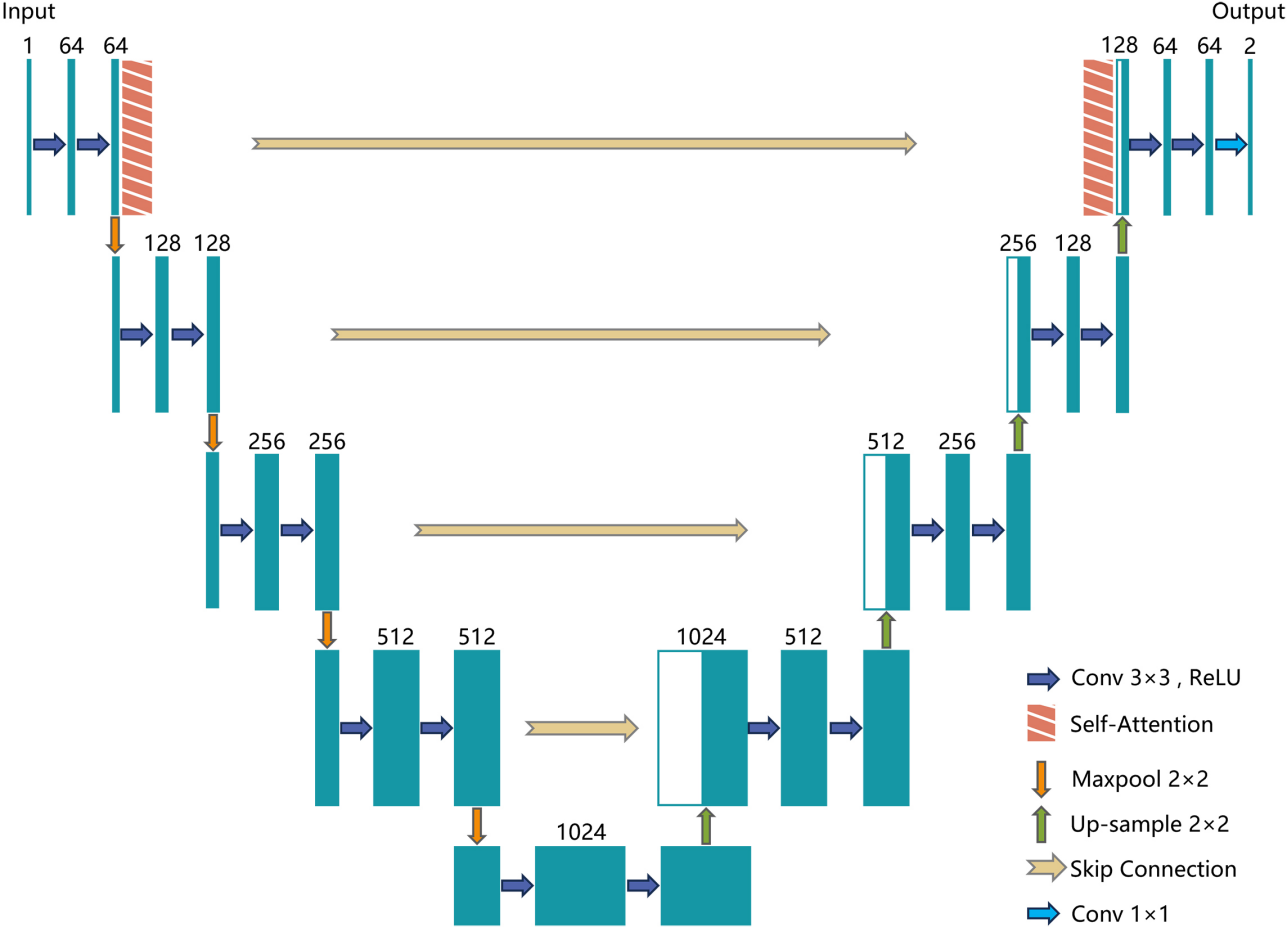
MGA-UNet model structure.

The output layer, usually a 1x1 convolutional layer, maps the feature maps to the required number of channels for the segmentation task. The number of output channels corresponds to the number of target classes. The architecture of the MGA-UNet model is illustrated in **Figure 4**.

The channel attention mechanism focuses on the relationships among different channels in the feature maps, generating a channel attention map that highlights the most significant channels. The process begins by taking a feature map of dimension H×W×C , where *H* is the height, *W* is the width, and *C* is the number of channels. Next, Global Average Pooling or Global Max Pooling is applied to transform the feature map into a channel descriptor, resulting in a 1×1×C vector. This descriptor is then passed through a fully connected layer (or convolutional layer) to produce a channel attention map of the same shape, 1×1×C . A Softmax or Sigmoid function is subsequently used to normalize this attention map, ensuring its values lie between 0 and 1. Finally, the normalized attention map is multiplied with the original feature map on a per-channel basis, thereby enhancing the representation of the more important channels in the final output.

The equations are expressed as follows:


Attention_map=σ(FC(Pooling(F)))

$$F^{\prime }=F⊗Attention\_map$$

where σ denotes the Sigmoid function, *FC* refers to the fully connected layer, Pooling represents the global pooling operation, and ⊗ denotes element-wise channel multiplication.

The spatial attention mechanism focuses on the correlation of spatial locations within the feature maps, generating a spatial attention map to highlight important regions in the image.

The spatial attention mechanism typically operates on a feature map of dimensions H×W×C , where *H* is the height, *W* is the width, and *C* is the number of channels. Through convolutional operations or similar transformations, this feature map is converted into a spatial attention map of dimensions H×W . A Softmax or Sigmoid function is then applied to normalize this attention map, ensuring its values fall between 0 and 1. Lastly, the normalized attention map is multiplied element-wise with the original feature map, effectively emphasizing crucial spatial locations.

The equations are expressed as follows:


Attention_map=σ(Conv(F))

$$F^{\prime }=F⊗Attention\_map$$

where *σ* denotes the Sigmoid function, Conv refers to the convolution operation, and ⊗ denotes element-wise channel multiplication.

### 2.6 Experimental setup

#### 2.6.1 Configuration information

To ensure a fair comparison, all experiments were conducted in a consistent hardware and software environment. Both the DCGAN and MGA-UNet models were trained using the PyTorch framework. The detailed hardware and software configurations are provided in **Table 3**.

**Table 3:** Hardware and software configuration information.

Item	Parameters	Version
Hardware	CPU	Intel(R) Xeon(R) Platinum 8352V CPU @ 2.10GHz
	GPU	NVIDIA RTX 4090 24GB
	RAM	90GB
Software	Python	3.12
	Pytorch	2.3.0
	CUDA	12.1
	CUDNN	8.9.1

#### 2.6.2 Training configuration

To enhance training efficiency, the drone-captured images were first stitched together into larger images and then uniformly processed into false-color images using multispectral data. Since drone images are too large to be processed directly by machine learning models, we divided them into smaller sub-images, each with dimensions of approximately 512 × 512 pixels.

Following traditional and generative model-based data augmentation techniques, a total of 9,972 images were used for training. The dataset consists of 3,348 diseased tree samples and 6,072 healthy tree samples. The data were randomly split into training, validation, and test sets in a 7:2:1 ratio. The dataset is divided into 6,980 training images, 1,994 validation images, and 998 test images.

The learning rate was adjusted using the Adam Optimizer. Adam (Adaptive Moment Estimation) is a widely adopted gradient descent algorithm in deep learning, known for its ability to handle sparse gradients and non-stationary targets (Kingma, 2014). Based on preliminary tests, the training parameters were set as follows: the entire training process spanned 100 epochs, balancing efficiency and accuracy. The batch size was set to 8, optimizing GPU utilization. Momentum was set to 0.9. The detailed training configuration is provided in **Table 4**.

**Table 4:** Detailed training configuration.

Parameters	Value
momentum	0.9
batch size	8
epoch	100
learning rate	Adam

#### 2.6.3 Evaluation metrics

To provide a comprehensive assessment, several evaluation metrics were employed, including mIoU (Mean Intersection over Union), mPA (Mean Pixel Accuracy), Precision, and Recall.

mIoU is a common metric in image segmentation tasks, evaluating model performance by measuring the overlap between the predicted region and the actual region. The IoU for each category is calculated as the intersection of the predicted and actual regions ( |Predicted Region∩Actual Region| )divided by the union of these regions ( |Predicted Region∪Actual Region| ). The mIoU is the average IoU across all categories.


$$IoU=\frac{\left|Predicted\;Region\cap Actual\;Region\right|}{\left|Predicted\;Region\cup Actual\;Region\right|}$$

$$mIoU=\frac{1}{N}\sum_{i=1}^{N}IoU_{i}$$

where *N* represents the total number of classes, and $IoU_{i}$ represents the IoU value for class.

mPA evaluates pixel accuracy by first calculating the accuracy for each category and then averaging across all categories.


$$PA_{C}=\frac{TP_{C}}{TP_{C}+FN_{C}}$$

$$mPA=\frac{1}{N}\sum_{c=1}^{N}PA_{C}$$

where $TP_{C}$ represents the number of true positive pixels for class. $FN_{C}$ represents the number of false negative pixels for class *c*. *N* represents the total number of classes. $PA_{C}$ denotes the pixel accuracy for class *c*.

Precision measures the accuracy of the model’s positive predictions, making it especially relevant for imbalanced datasets. Precision is calculated as the number of true positive predictions divided by the sum of true positives and false positives.


$$Precision=\frac{TP}{TP+FP}$$

where *TP* represents the number of true positive samples. *FP* represents the number of false positive samples.

Recall complements Precision by indicating the proportion of actual positives correctly identified by the model. Recall is calculated as the number of true positive predictions divided by the sum of true positives and false negatives.


$$Recall=\frac{TP}{TP+FN}$$

where *TP* (True Positives) represents the number of correctly predicted positive samples. *FN* (False Negatives) represents the number of samples that are actually positive but were incorrectly predicted as negative.

## 3 Results

This study systematically examined the differences between using multispectral and high-resolution image datasets. It also explored the benefits of employing generative model-based data augmentation to enhance the dataset and analyzed the impact of attention mechanisms on image segmentation performance. Experiments were conducted across various data and algorithm combinations: the first group utilized high-resolution images, the second group used color-synthesized multispectral images, and the third group employed images augmented by generative models. Multispectral images consist of datasets captured simultaneously by a multispectral camera and a high-resolution camera at the same spatial scale. In this study, we compared the classification performance between these different image datasets. The specific experimental results are shown in **Table 5**.

**Table 5:** Specific experimental results.

Evaluation Metrics	Classification	High definition image	Multispectral image	Data Augmentation
IoU	mIoU	0.59	0.81	0.92
	**sick**	**0.35**	**0.73**	**0.89**
	health	0.48	0.76	0.91
	background	0.93	0.94	0.95
PA	mPA	0.73	0.89	0.96
	**sick**	**0.4**	**0.82**	**0.94**
	health	0.82	0.89	0.96
	background	0.97	0.96	0.97
Precision	sum	0.75	0.89	0.96
	**sick**	**0.76**	**0.87**	**0.95**
	health	0.53	0.83	0.94
	background	0.96	0.97	0.98
Recall	sum	0.73	0.89	0.96
	**sick**	**0.4**	**0.82**	**0.94**
	health	0.82	0.89	0.96
	background	0.97	0.96	0.97

The category name "sick," marked in bold in the table, represents the values of each evaluation metric for diseased fruit tree regions of interest infected with Citrus Huanglongbing after processing by the image segmentation model.

### 3.1 Comparison between high-resolution images and multispectral images

The drone used in this study was equipped with both a high-resolution camera and a multispectral camera, allowing simultaneous capture of high-resolution images and corresponding multispectral Images during a single flight. The high-resolution images were stitched into larger mosaics and then uniformly cropped into segments suitable for training. These images, consisting of three color channels (R, G, B), capture visual information similar to that perceived by the human eye but offer limited informational depth. In the context of early Citrus Huanglongbing (HLB) detection, the subtle pathological features of infected citrus trees are challenging to discern using high-resolution images alone. The focus of this study was on the recognition accuracy of positive samples, specifically the identification rate of infected trees. For high-resolution images, the model achieved a mean Intersection over Union (mIoU) of 0.35, mean Pixel Accuracy (mPA) of 0.4, Precision of 0.76, and Recall of 0.4. These relatively low recognition rates indicate that high-resolution imagery is insufficient for the early detection of HLB.

**Figure 5:**
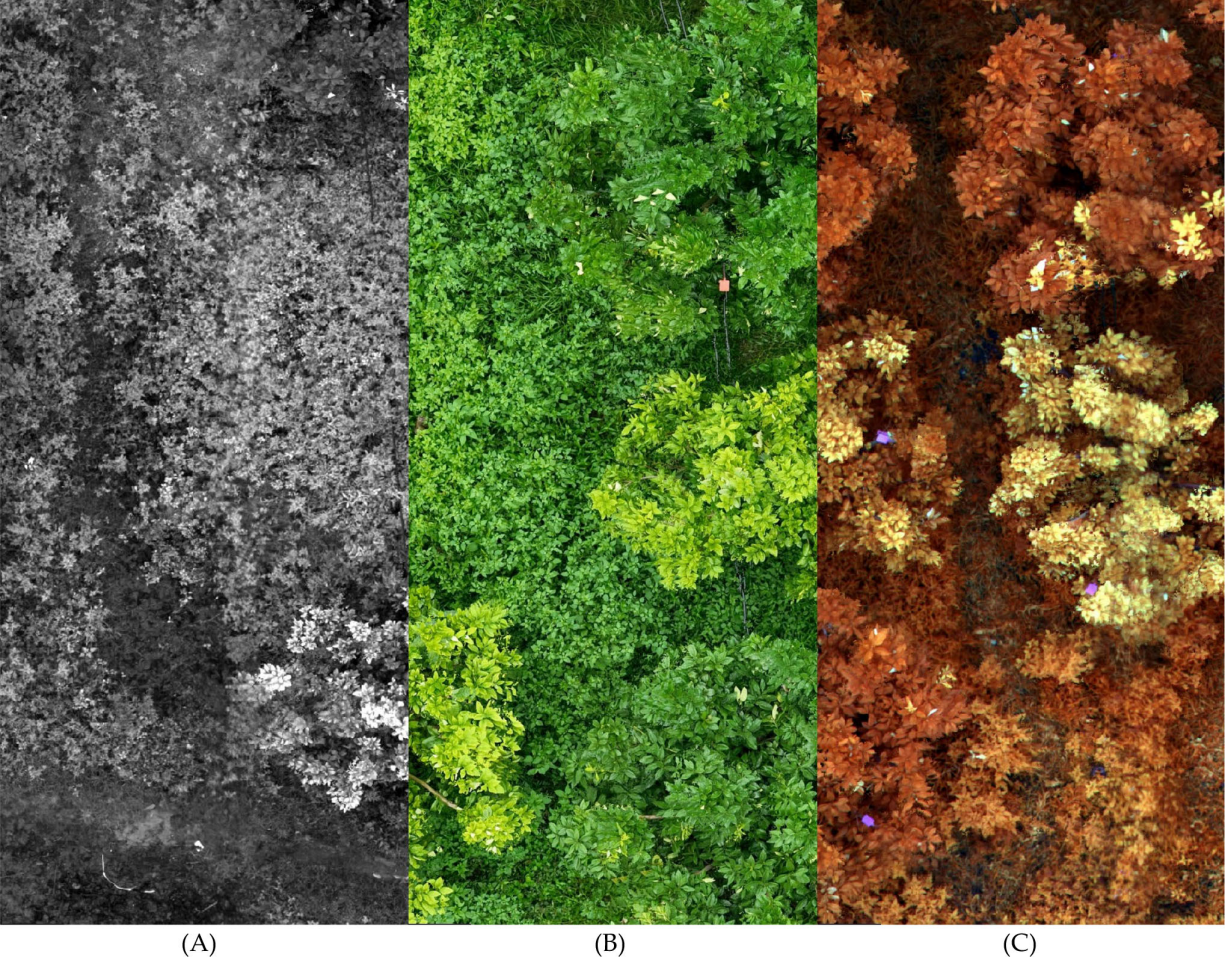
This figure illustrates the differences between a multispectral image, a high-resolution image, and a false-color composite image. In the figure, image **(A)** rep-resents the grayscale image of a single band from the multispectral image. Image **(B)** is a high-resolution image composed of three RGB bands. Image **(C)** is a false-color composites image created by combining multiple bands from the multispectral image.

**Figure 6:**
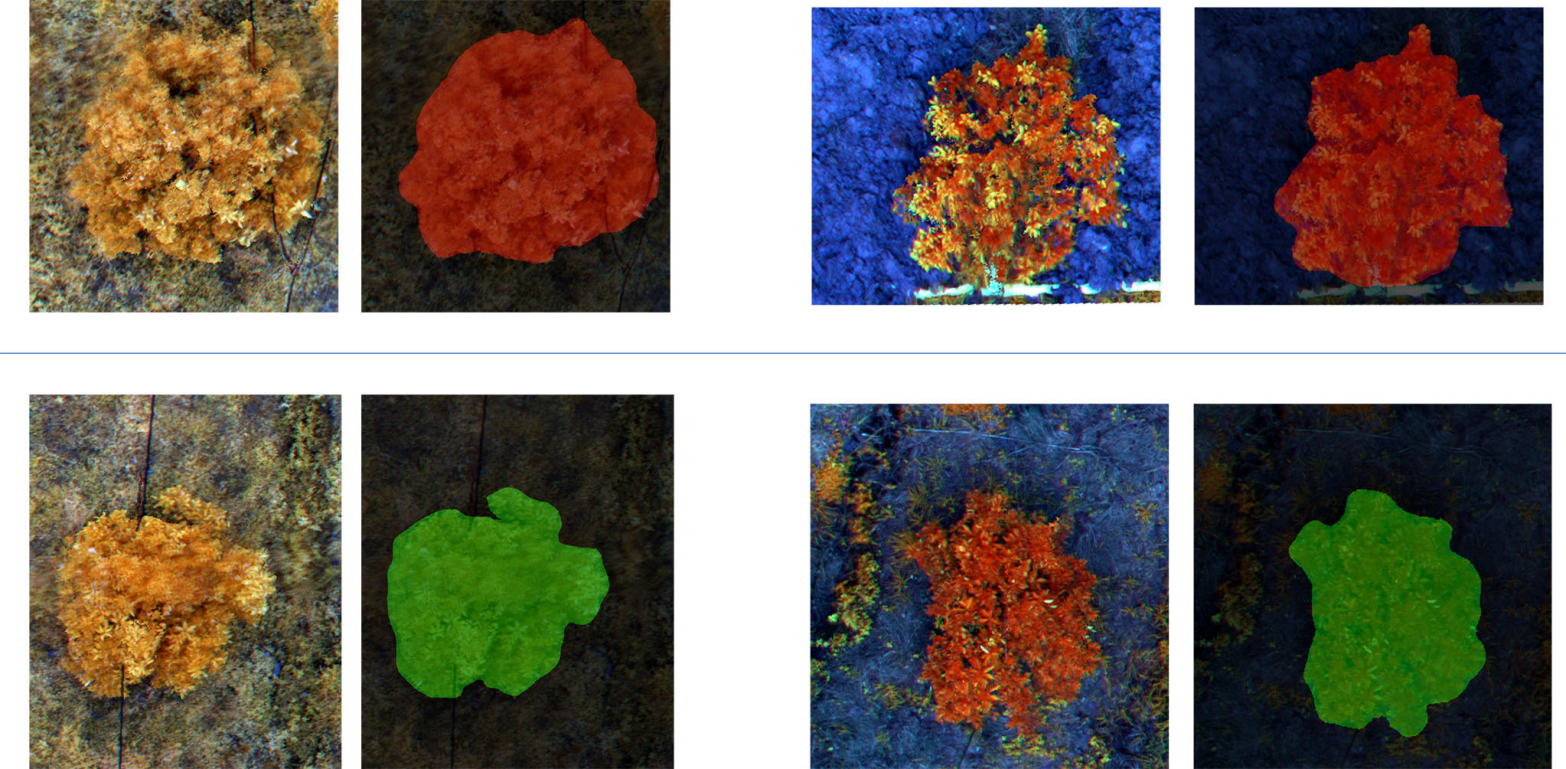
This figure illustrates the classification results of multispectral images segmented into small patches by the MGA-UNet model. Each displayed image contains only a single sample. The two images in the first row represent samples identified as “Citrus disease: HLB” indicated by the red pixel mask overlays. The two images in the second row represent samples classified as “Healthy,” highlighted by the green pixel mask overlays.

**Figure 7:**
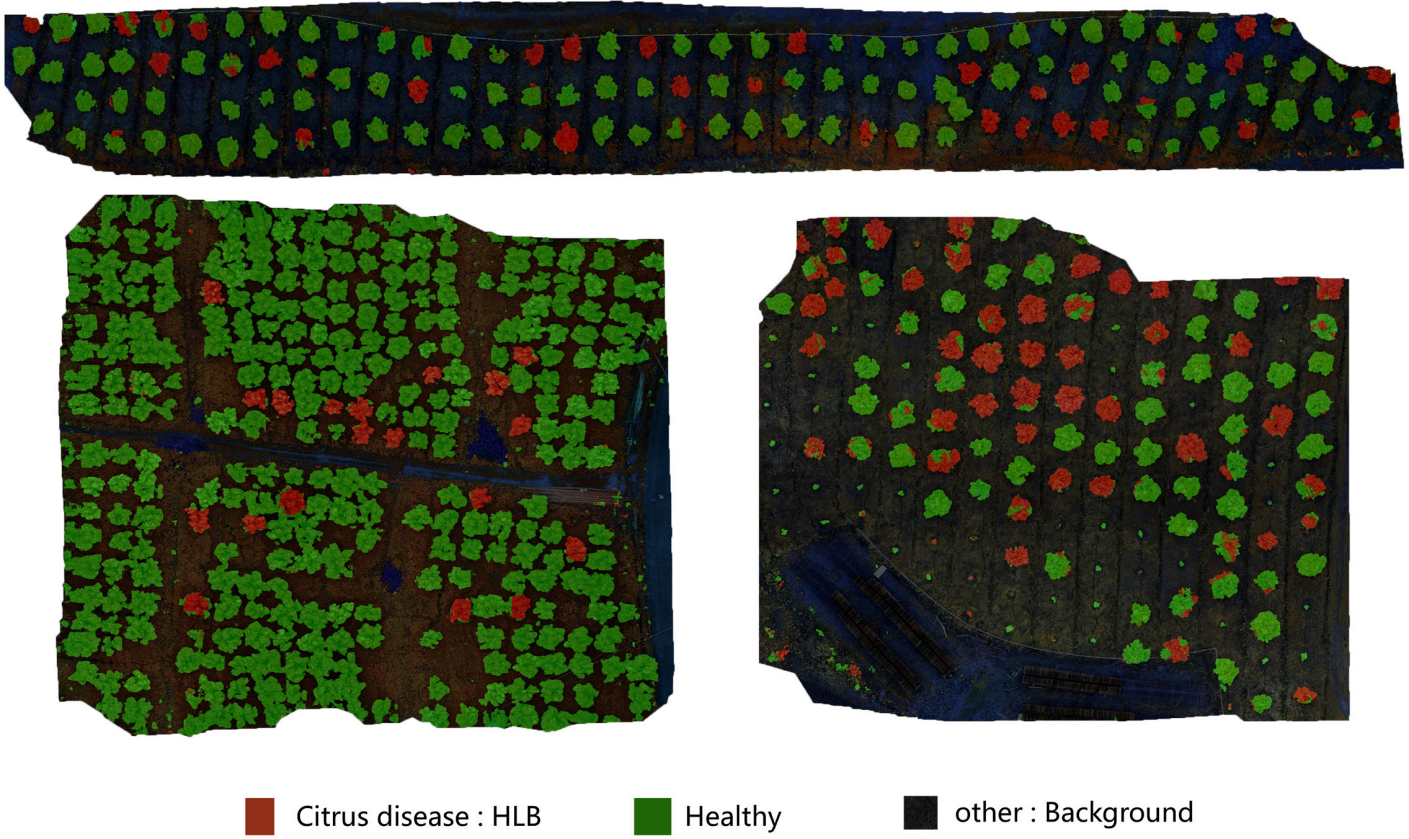
This figure demonstrates the direct classification results of large-scale UAV-acquired images using the MGA-UNet model. Trees partially covered with red pixel areas are identified as “Citrus disease: HLB” even if other regions of the same tree exhibit green pixels. This phenomenon occurs because, during the early stages of HLB infection, pathological symptoms may not yet manifest uniformly across all leaves of a single tree.

To enhance the model’s ability to detect early-stage HLB features, multispectral images were employed as the primary dataset. These images, captured by the drone’s multispectral camera, included four spectral bands: Red Edge (RE), Red (R), Near-Infrared (NIR), and Green (G). The multispectral images were then color-synthesized into false-color composites images for training. A comparison between a multispectral image, a high-resolution image, and a false-color composite image is provided in the **Figure 5**. For the detection of diseased trees using multispectral images, the model achieved an mIoU of 0.73, mPA of 0.82, Precision of 0.87, and Recall of 0.82. The recognition accuracy improved significantly when switching from high-resolution to multispectral images. This result demonstrates that multispectral imaging is more suitable for the early detection of HLB, offering greater accuracy compared to high-resolution images. The visualization of the recognition results for the multispectral images, which were segmented into individual sample images, is presented in **Figure 6**. Meanwhile, this study also provides a large-scale map-based visualization of the hyperspectral images, as illustrated in **Figure 7**.

### 3.2 Impact of generative model-based data augmentation

A key challenge in the early detection of HLB lies in the relatively small proportion of diseased samples within the dataset. During the initial stages of HLB infection, the virus spreads slowly, resulting in significantly fewer diseased samples compared to healthy ones, which in turn leads to a pronounced dataset imbalance. This imbalance often induces model overfitting, adversely affecting training outcomes. Moreover, simply reducing the number of healthy samples to address this imbalance shrinks the overall dataset, complicating the statistical analysis of HLB spread trends.

To mitigate these issues, this study employed a generative adversarial network (DCGAN) to augment the dataset by generating synthetic images of HLB-infected trees. DCGAN learns the distinguishing features of diseased images from the existing dataset and creates novel synthetic images that closely resemble authentic diseased samples yet remain unique. This approach effectively alleviates dataset imbalance while preserving statistical rigor, offering a marked advantage over conventional augmentation techniques.

Nevertheless, DCGAN has certain limitations and areas for further refinement. First, DCGAN requires a sufficiently large training dataset; it may perform suboptimally on very small datasets. Second, although DCGAN can produce high-quality diseased samples, the resolution of these generated images is often relatively low, which may limit subsequent model performance. Future research may explore more advanced generative algorithms or enhanced hardware to increase image resolution and quality. Additionally, generated synthetic data should not be recycled as training inputs in the same generative model, as doing so may cause model contamination, producing progressively distorted outputs (Wenger, 2024).

Despite these drawbacks, generative models such as DCGAN offer unique benefits for data augmentation by reducing model overfitting and bolstering robustness. In HLB detection, minor distortions in structural details—problematic in other applications like human facial recognition—are less critical. While multispectral imaging outperformed other approaches in this study, its higher acquisition and processing costs may limit large-scale commercial adoption. Consequently, practical applications must balance the expense of data acquisition with the desired detection accuracy.

**Figure 8:**
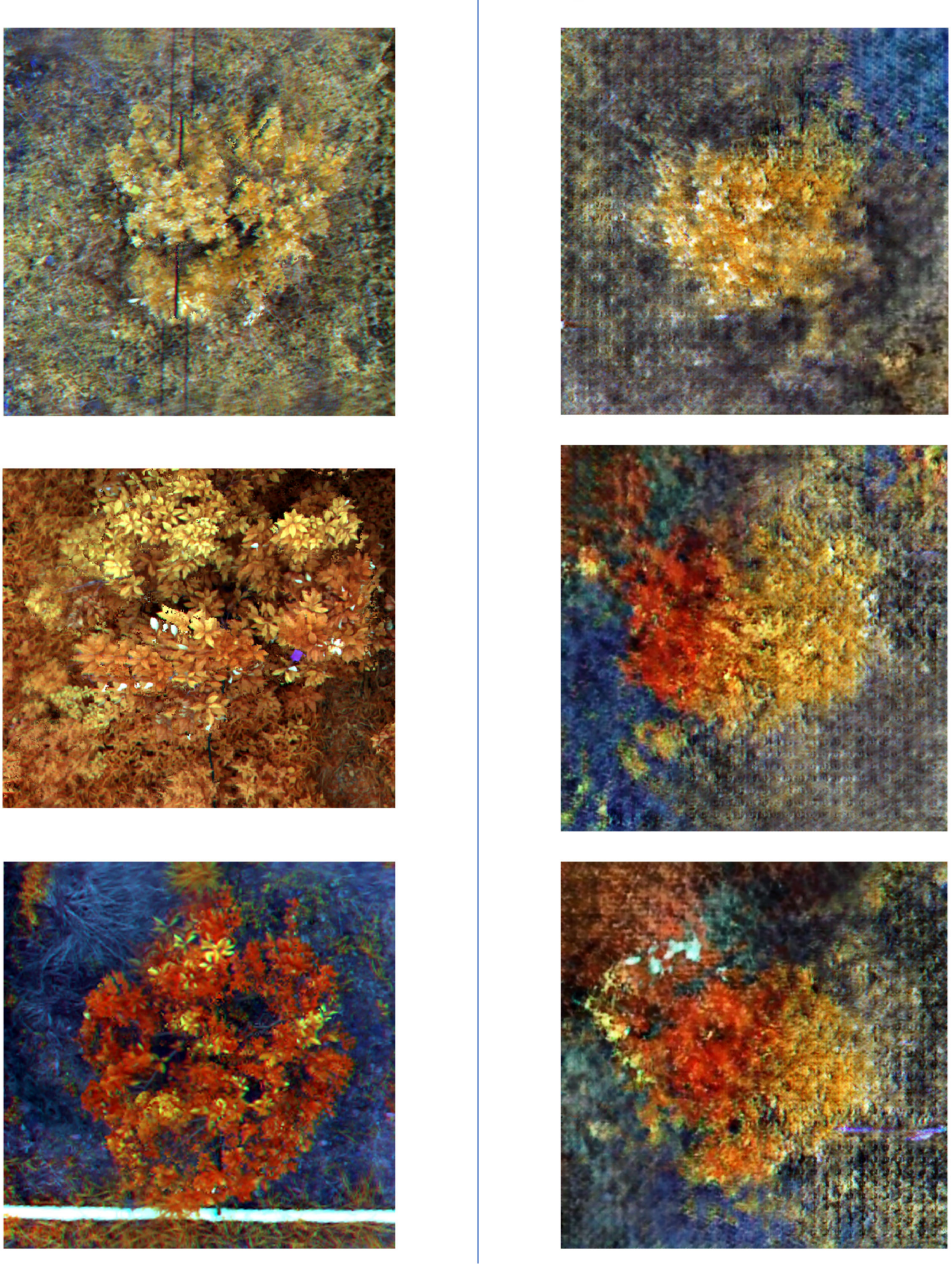
This figure presents a comparison between real and generated images. The three images on the left show real images that have undergone color composition processing, while the three images on the right display images generated by the DCGAN model after being trained on a dataset of real images.

Overall, the use of a generative model for data augmentation in the HLB dataset proves to be a viable solution. For the identification of diseased trees, the model achieved an mIoU of 0.89, mPA of 0.94, Precision of 0.95, and Recall of 0.94. Compared to the recognition accuracy prior to the application of generative model-based data augmentation, these results show a notable improvement, significantly enhancing the model’s robustness. The comparison of the real images and generated images is shown in **Figure 8**.

## 4 Discussion

The study’s results highlight the substantial benefits of integrating multispectral imaging with deep learning algorithms for the early detection of Citrus Huanglongbing (HLB). A comparative analysis between high-resolution and multispectral images shows that multispectral images consistently outperform high-resolution images in key metrics, including mIoU, mPA, Precision, and Recall. This is especially evident in the identification of diseased trees. These findings underscore the efficacy of multispectral imaging in capturing early pathological features of plants. Unlike high-resolution images, multispectral images offer richer spectral information across four bands—Red Edge (RE), Red (R), Near-Infrared (NIR), and Green (G)—which more effectively highlight the subtle early indicators of HLB. Consequently, multispectral imaging significantly enhances the accuracy of HLB detection, especially in the early stages where symptoms are less pronounced.

Moreover, the application of the Generative Adversarial Network DCGAN for data augmentation yielded notable improvements. The DCGAN-generated images increased the proportion of diseased samples, effectively addressing dataset imbalance and boosting performance metrics related to the identification of diseased trees. Compared to traditional data augmentation techniques, generative models not only diversify the dataset but also bolster model robustness, leading to overall performance enhancements. While the generated images may introduce slight distortions in fine structures, these do not significantly impact the model’s performance in plant disease detection. This suggests that DCGAN holds considerable promise for overcoming data augmentation challenges in plant recognition.

The MGA-UNet model developed in this study further advanced image segmentation performance. By incorporating channel and spatial attention mechanisms into the U-Net architecture, MGA-UNet more effectively extracts and leverages detailed information from multispectral images. This improvement is particularly focused on identifying diseased regions. This refinement has led to improved segmentation accuracy. The experimental outcomes demonstrate that the MGA-UNet model is both applicable and effective for the early detection of HLB.

By integrating multispectral imaging, DCGAN-based data augmentation, and the MGA-UNet model, this study offers a robust solution for the early detection of HLB. This approach not only enhances disease recognition accuracy but also provides valuable insights for future research in plant disease detection.

## 5 Conclusions

Citrus Huanglongbing (HLB) poses substantial challenges for early detection due to its subtle pathological features. In this study, multispectral images captured by drones were used as the primary dataset, and an optimized MGA-UNet model was proposed for image segmentation and recognition. By integrating channel and spatial attention mechanisms into the skip connections of a U-Net architecture, the model achieved more effective feature retention and mapping, leading to superior performance in detecting and monitoring HLB.

To address the imbalance in the dataset, a Generative Adversarial Network (DCGAN) was employed to generate additional HLB-infected samples, thereby improving the reliability of model training. While DCGAN effectively produced usable synthetic data, its resolution limitations warrant exploration of more advanced generative methods or improved hardware for higher-quality images. Furthermore, although multispectral imaging demonstrated notable advantages, its cost may limit large-scale adoption, necessitating a careful balance between detection accuracy and practicality.

Experimental results showed that the proposed model achieved an mIoU of 0.89, an mPA of 0.94, a Precision of 0.95, and a Recall of 0.94. These findings underscore the potential of deep learning in conjunction with drone-acquired multispectral data to facilitate the early detection and identification of HLB, thereby partially reducing the reliance on manual inspections.

## Data Availability

The original contributions presented in the study are included in the article/supplementary material. Further inquiries can be directed to the corresponding authors.
